# First–Second-Trimester Dietary Inflammatory Index and Anemia Risk in the Third Trimester: A Prospective Cohort Study

**DOI:** 10.3390/nu17111938

**Published:** 2025-06-05

**Authors:** Cong Huang, Zhitan Zhang, Junwei He, Zixin Zhong, Yuxin Ma, Xun Huang, Fan Xia, Hongzhuan Tan, Jing Deng, Mengshi Chen

**Affiliations:** 1 Department of Epidemiology and Health Statistics, Xiangya School of Public Health, Central South University, No. 172 Tongzipo Road, Yuelu District, Changsha 410013, China; huangcc2000@163.com (C.H.); 18661893668@163.com (Z.Z.); hjw001007@163.com (J.H.); 13574652893@163.com (Z.Z.); 226911023@csu.edu.cn (Y.M.); huangxunxun66@163.com (X.H.); 226901005@csu.edu.cn (F.X.); tanhz@mail.csu.edu.cn (H.T.); jingdeng@csu.edu.cn (J.D.); 2 Hunan Provincial Key Laboratory of Clinical Epidemiology, Xiangya School of Public Health, Central South University, No. 172 Tongzipo Road, Yuelu District, Changsha 410013, China

**Keywords:** pro-inflammatory diet, pregnancy, iron supplementation, Chinese

## Abstract

**Objectives:** Dietary conditions are closely related to maternal health. This study aims to investigate the causal relationship between the first–second-trimester Dietary Inflammatory Index (DII) and developing anemia in the third trimester. **Methods:** This prospective cohort study comprised 545 pregnant women, with dietary data assessed via a semi-quantitative food frequency questionnaire (FFQ). Hemoglobin levels were obtained by hospital laboratory tests and used to diagnose anemia. Multivariable logistic regression models—adjusted for baseline serum iron, age, pre-pregnancy body mass index (BMI), occupation, education, history of adverse pregnancy outcomes, parity, serum iron, passive smoking exposure, and iron supplementation use during pregnancy—were employed to evaluate the relationships between the first-trimester DII, second-trimester DII, first–second-trimester average DII, and third-trimester anemia. **Results:** After multivariable adjustment, the first–second-trimester average DII in the pro-inflammatory diet group demonstrated a 3.73-fold elevated risk of third-trimester anemia compared to the anti-inflammatory diet group (Odds Ratio [OR] = 3.73, 95% Confidence Interval [CI]: 1.50–9.25). **Conclusions:** Pro-inflammatory dietary patterns during pregnancy exhibit a significant correlation with developing third-trimester anemia. This study demonstrates that reducing dietary pro-inflammatory components through prenatal nutrition programs may lower third-trimester anemia risk. Notably, this study carries potential risks of bias, including self-reporting bias in dietary data and incompletely controlled confounding factors (such as unmeasured biomarkers).

## 1. Introduction

Anemia, the most common hematologic disorder, is an important health concern globally and is more prevalent in the pregnant population. Globally, 36.8% of pregnant women are affected by anemia, marked by significant geographical disparities [1], where developing countries exhibit substantially higher rates than developed nations [2,3]. The prevalence of anemia in pregnancy among Chinese pregnant women is reported to be 23.5% [4]. Previous studies [5,6] have identified an elevated risk of anemia in the third trimester of pregnancy compared to earlier pregnancy stages. Anemia in the third trimester has been confirmed to be significantly associated with elevated risks of postpartum hemorrhage, preterm birth, and other adverse obstetric outcomes [7,8,9], posing substantial threats to maternal and neonatal health.

Anemia in pregnancy is primarily attributed to the dysregulation of iron metabolism, encompassing insufficient iron intake, suboptimal dietary patterns, and low compliance with iron supplementation [10,11]. Nutritional deficiencies, particularly an inadequate intake of protein and vitamins during pregnancy, further increase the risk of developing anemia [12,13]. Iron supplementation during pregnancy serves as a critical strategy for preventing gestational anemia [14]. Contemporary clinical recommendations [15] advocate intermittent iron–folic acid supplementation for pregnancies without anemia, while screening-diagnosed anemia cases necessitate intensified therapeutic protocols involving additional iron intake during pregnancy. Marked disparities in iron supplementation rates exist among pregnant women across different areas of China [4,16]. The iron supplementation rate in rural Northwest China (5.4%) is markedly lower than that in developed regions [17]. However, even with standardized iron supplementation, a subset of pregnant women remains susceptible to anemia [18]. This suggests the existence of mechanisms beyond single nutrient deficiencies [19], including inflammation [20].

The role of inflammation in the incidence and persistence of anemia has attracted growing interest [21]. Inflammatory responses impair erythrocyte homeostasis by interfering with erythropoiesis and regulatory pathways, potentially contributing to heterogeneous anemia subtypes [22,23]. Dietary components are closely associated with inflammatory processes in humans. Red meat (beef, lamb, etc.) is rich in highly bioavailable heme iron [24,25], which is critical in combating iron deficiency anemia and is prioritized in maternal nutrition protocols for prophylactic iron supplementation [26]. However, some studies suggest that red meat may also be associated with elevated serum levels of inflammatory mediators [27,28]; this nutritional paradox triggers a reconsideration of dietary intervention strategies. Therefore, a further understanding of the relationship between inflammation and anemia can provide a basis for a more scientific approach to the dietary prevention of anemia during pregnancy.

The Dietary Inflammatory Index (DII), which quantifies the inflammatory potential of dietary intake [29], offers novel insights into exploring diet–anemia relationships during pregnancy. The DII calculates composite scores through the weighted assessment of 45 dietary components based on their pro-/anti-inflammatory effects derived from the established literature, with higher scores indicating pro-inflammatory diets and lower scores reflecting anti-inflammatory patterns [30]. While the DII has been extensively applied in chronic disease research [31], its utilization in maternal health remains limited. Existing studies in pregnant populations predominantly focus on gestational diabetes and preeclampsia [32,33]. However, studies examining the relationship between the DII and anemia remain scarce, and what is particularly lacking is robust evidence of a causal relationship from prospective cohort studies [30].This prospective cohort study assesses the longitudinal relationship between the first–to–second-trimester DII and subsequent third-trimester anemia risk, with temporality supporting possible causal pathways. The fundamental hypothesis of this study is that a cause–effect relationship exists between the DII during the first–to–second-trimester and third-trimester anemia and that higher DII scores (reflecting a pro-inflammatory diet) serve as a risk factor for anemia in the third trimester. The results may inform the development of anti-inflammatory dietary interventions, potentially reducing the risk of anemia in pregnancy and improving pregnancy outcomes.

## 2. Materials and Methods

### 2.1. Study Design and Participants

This study recruited women with singleton pregnancies from a maternal–child healthcare facility in China between 2017 and 2019. The inclusion criteria for pregnant women included the following: registered in obstetrics departments of collaborating hospitals with complete standardized antenatal records; aged 18–45 years; and cognitively competent to provide written informed consent for voluntary study participation.

**Figure 1:**
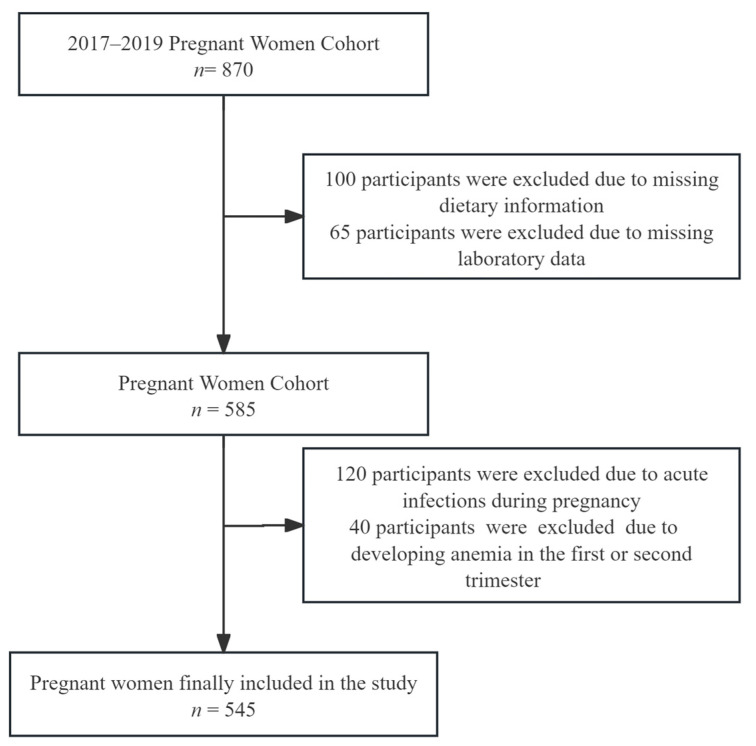
Inclusion and exclusion flow chart.

From 870 initially recruited participants, 545 eligible subjects were included after excluding those with missing key data, acute infections during pregnancy, antibiotic use (participants with acute infections were excluded to eliminate confounding effects [34]), or anemia developed before the third trimester (Figure 1). The study protocol was authorized by the Medical Ethics Committee of Hunan Provincial Maternal and Child Health Care Hospital (No. EC201624), with written informed consent collected from all participants.

### 2.2. Sample Size Calculation

This study is a prospective cohort design. Based on the existing literature, including multiple studies linking the pregnancy-related Dietary Inflammatory Index (DII) to adverse pregnancy events [35,36,37], we hypothesized a relative risk (RR) of 1.5 with a population exposure rate of 23.8% [4]. Using a significance level of α = 0.05 (two-tailed) and β = 0.1, the target sample size was calculated as 308 participants to achieve adequate statistical power, according to the following epidemiological formula (Formula (1)). This study involved 545 participants, fulfilling the research requirements.

(1) $$N=\frac{{\left(Z_{\alpha }\cdot \sqrt{2\cdot p\cdot q}+Z_{\beta }\cdot \sqrt{p_{0}\cdot q_{0}+p_{1}\cdot q_{1}}\right)}^{2}}{{\left(p_{0}-p_{1}\right)}^{2}}$$

### 2.3. Survey of Participants’ Information 

Baseline characteristics were collected through structured questionnaires at the time of pregnancy registration, including age, occupation, education, pre-pregnancy body mass index (BMI), parity (counting only full-term pregnancies), and adverse pregnancy history (including spontaneous abortion, dystocia, postpartum hemorrhage, etc.). Data on passive smoking, alcohol use, and supplement intake (e.g., calcium, iron, folic acid, vitamins) were obtained via questionnaires during follow-ups in the first and second trimesters. Baseline serum iron levels and hemoglobin values from the first to the third trimester were obtained from hospital laboratory tests.

### 2.4. Dietary Assessment

Dietary intake was evaluated via a semi-quantitative FFQ during the first (11–13 weeks) and second trimesters (24–27 weeks) of pregnancy. The FFQ comprised 13 food categories: cereals, tubers, vegetables, fruits, poultry and livestock meats, seafood, freshwater products, dairy products, soybean products, nuts, edible oils, beverages, and alcoholic beverages. The FFQ included the following response options for intake frequency: ‘Once or more per day’, ‘Once or more per week’, ‘Once or more per month’, and ‘Never’, along with data on the consumption frequency and serving size per occasion. Each dietary questionnaire was self-administered by the participants. In addition, based on the standardized nutritional labeling of commercially available supplements chosen by participants, we calculated supplemental nutrient intakes after unit normalization. These supplemental values were then combined with dietary sources to compute the DII score.

### 2.5. DII Calculation

The DII is a validated tool with established construct validity and computational methods [30]. Using data from FFQ-administered dietary assessments and participants’ nutritional supplement records, the daily intakes of 26 components were quantified. These comprised energy, protein, carbohydrates, fats, cholesterol, saturated fatty acids (SFAs), monounsaturated fatty acids (MUFAs), polyunsaturated fatty acids (PUFAs) (*n*-3 and *n*-6 subtypes), dietary fiber, vitamins (A, C, D, E, B1, B2, B3 [niacin], B6, B9 [folate], B12 [cobalamin]), beta-carotene, and minerals (iron, zinc, selenium, magnesium). For each participant, dietary parameters were standardized as Z-scores relative to a global reference database, calculated using the following formula: Z-score = (daily intake − mean)/standard deviation (SD) for each component. To correct for right skewness, the Z-score underwent a centered proportion transformation. Each proportion was weighted by its food parameter effect score, and the cumulative DII score represented the summation of all weighted dietary parameters.

The diet is unstable in the first trimester due to pregnancy reactions (e.g., vomiting) and stabilizes in the third trimester. The two-stage average score of the DII is a truer reflection of the dietary habits of pregnant women during pregnancy. In addition, because of the lag in iron metabolism [38], the development of anemia requires some time for the accumulation of nutritional imbalances. If only the mid-pregnancy diet is analyzed, the effect of the early-pregnancy diet on anemia may be overlooked. Therefore, both DII scores from the first and second trimesters and the averaged DII across these two trimesters were included as exposure variables in the analysis.

Participants were categorized into three distinct groups according to the DII score distribution (25th and 75th percentiles): those with DII scores below the 25th percentile were classified as the anti-inflammatory diet group; individuals with DII scores above the 75th percentile were designated as the pro-inflammatory diet group; and participants scoring between the 25th and 75th percentiles comprised the intermediate group.

### 2.6. Diagnostic Criteria and Variable Definitions

(1) Hemoglobin (Hb): Hemoglobin levels are obtained by hospital laboratory tests. WHO standards [31] were applied for anemia diagnosis, using trimester-specific Hb thresholds: <110 g/L (first/third trimesters) and <105 g/L (second trimester) [39].
(2) BMI: According to Chinese national guidelines [40], BMI was categorized into categories defined as non-overweight (BMI < 24 kg/m^2^) and overweight (BMI ≥ 24.0 kg/m^2^).
(3) Passive smoking exposure: Passive smoking exposure during pregnancy was defined as self-reported regular exposure to secondhand smoke in household or occupational settings, dichotomously categorized based on questionnaire responses (yes/no).

### 2.7. Covariates

This study adjusted for key covariates to strengthen the causal inference between the DII and anemia, including age (<30 years, ≥30 years), pre-pregnancy BMI (<23.9 kg/m^2^, ≥24.0 kg/m^2^), occupation (employed, unemployed), education (below high school, high school and above), history of adverse pregnancy (no, yes), parity (0, ≥1), baseline serum iron (SI), passive smoking exposure (yes/no), and iron supplementation use (yes/no).

### 2.8. Statistical Analysis

Data entry was conducted utilizing EpiData 3.1 software with dual independent data entry and cross-verification to ensure accuracy. Statistical analysis and plotting were performed using SPSS 26.0 and R 4.4.3. Normally distributed continuous variables (e.g., age, BMI) are presented as the mean ± SD, whereas non-normal data are expressed as the median (IQR). Categorical variables (e.g., occupation, parity, history of adverse pregnancy outcomes) are presented as percentages. Continuous variables adhering to a normal distribution were evaluated via parametric tests. Chi-square tests were used to compare the distribution of participant characteristics across DII tertiles and to assess the relationships between participant characteristics and third-trimester anemia. To address Type I error inflation, the Bonferroni correction was implemented for multiple intergroup comparisons

Multivariable logistic regression models were employed to evaluate the correlation between the gestational DII and third-trimester anemia and reported odds ratios (ORs) and 95% confidence intervals (CIs). Restricted cubic spline regression was used to analyze the dose–response trends between DII scores and anemia risk. The criterion for statistical significance was determined by a two-tailed *p*-value < 0.05.

## 3. Results

### 3.1. Baseline and Pregnancy Characteristics of Participants

This study enrolled 545 participants. The mean age was 29.50 ± 4.28 years, and the pre-pregnancy BMI averaged 20.77 ± 2.64 kg/m^2^. The baseline serum iron levels were 8.76 ± 0.85 μmol/L. Sociodemographic characteristics indicated that 87.3% of participants completed senior high school or higher education, 88.0% were employed, and 82.3% were primiparous. During pregnancy, 18.9% reported passive smoking exposure, and 42.2% received iron supplementation.

The first trimester DII scores extended from −4.38 to 3.81, with the median (25th percentile, 75th percentile) being 0.12 (−1.29, 1.45). The second-trimester DII scores extended from −4.84 to 3.91. The median and interquartile range (IQR) were 0.33 (−1.12–1.63). The First–second-trimester average DII scores extended from −3.25 to 3.50, with the median (25th percentile, 75th percentile) being 0.24 (−0.95, 1.17).

Table 1 reveals no significant differences in baseline or pregnancy characteristics across the DII groups.

### 3.2. Incidence of Third-Trimester Anemia

Of the 545 participants, 61 developed anemia during the third trimester, yielding an incidence rate of 11.19%, with all cases classified as mild anemia [4]. As shown in Table 2, baseline characteristic analysis revealed a significantly lower cumulative incidence of anemia in the third trimester among participants exposed to secondhand smoke during pregnancy compared to non-exposed individuals (*p* < 0.05). Stratified by first–second-trimester average DII groups, the cumulative incidence in the pro-inflammatory diet group was 15.16%, significantly exceeding the 5.19% incidence in the anti-inflammatory diet group (Bonferroni-adjusted *p* = 0.21).

### 3.3. Relationship Between First–Second-Trimester DII and Anemia in Third Trimester

We applied logistic regression to evaluate the longitudinal association of the first–second-trimester DII with later third-trimester anemia (Table 3). Adjusted for confounders including age, educational attainment, parity, secondhand smoke exposure, and iron supplementation, the first–second-trimester average DII in the pro-inflammatory diet group exhibited a 3.73-fold elevated risk relative to the anti-inflammatory diet group (OR = 3.73, 95% CI: = 1.50–9.25). In addition, the second-trimester DII scores exhibited a statistically significant association with an increased risk of third-trimester anemia after stepwise adjustment for confounders, with consistent effect magnitudes across all models (*p* < 0.05). Although first-trimester DII scores demonstrated a positive trend toward anemia risk (OR = 1.15, 95% CI: 0.99–1.34), statistical significance was not attained for this association.

**Figure 2:**
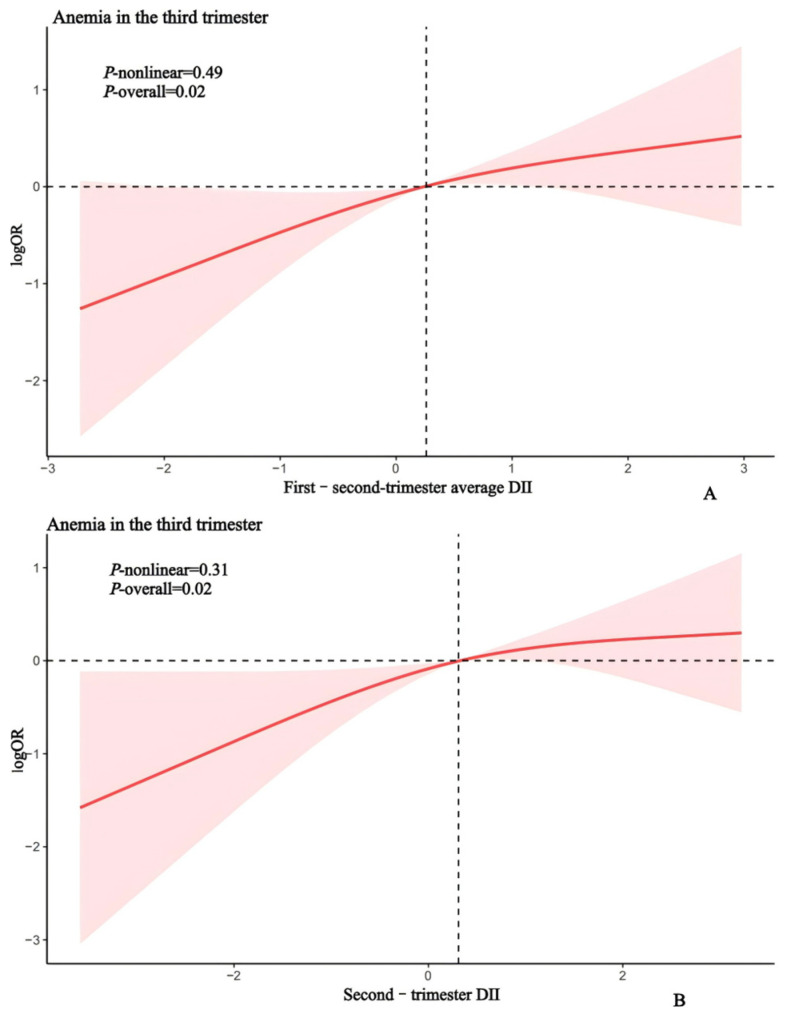
Sample regression model for second-trimester DII (**A**), first–second-trimester average DII (**B**), and third-trimester anemia. Restricted cubic spline regression was adjusted for age, pre-pregnancy BMI, education, occupation, parity, serum iron, and a history of adverse pregnancy outcomes, passive smoking exposure and iron supplementation.

Restricted cubic spline regression further revealed a significant dose-dependent increase in anemia risk with rising second-trimester and first–second-trimester average DII scores (Figure 2).

### 3.4. Stratified Analysis of Iron Supplementation, DII, and Anemia Risk

Given the established association between anemia and iron supplementation, stratified logistic regression analyses were conducted based on iron supplementation status. In the non-supplemented group (*n* = 315), a 1-unit rise in the second-trimester DII corresponded to a 41.3% increase in anemia risk (adjusted OR = 1.40, 95% CI= 1.12–1.78, *p*= 0.004), while a 1-unit increase in the first–second-trimester average DII corresponded to a 62.3% higher risk (adjusted OR = 1.62, 95% CI= 1.19–2.22, *p* = 0.002). Conversely, no significant associations were observed between the DII and anemia in the iron-supplemented group (*n* = 230) for either the second-trimester DII (adjusted OR = 1.12, *p* = 0.321) or average DII (adjusted OR = 1.19, *p* = 0.199) (Table 4).

Furthermore, interaction effects between the DII and iron supplementation status were assessed via Wald tests, with no statistical significance observed across all trimesters (*p* > 0.05).

## 4. Discussion

### 4.1. Main Findings Compared to Previous Studies

This prospective study systematically examined whether first–second-trimester DII is an influential factor in the development of third-trimester anemia using longitudinal data. It revealed that a pro-inflammatory dietary pattern (higher first–second-trimester average DII score) independently increases the risk of third-trimester anemia (OR = 3.73), with a stronger association observed among pregnant women who did not use iron supplements.

While numerous studies have evaluated the importance of dietary interventions in preventing or treating anemia, traditional research on pregnancy-related anemia has primarily focused on iron supplementation and the correction of single-nutrient deficiencies [41,42,43,44]. For example, strategies to increase iron intake through foods rich in iron (e.g., meat, fish, legumes, and leafy greens) [45,46] or isolated supplementation with vitamin A or other micronutrients [47,48] may improve maternal hemoglobin levels and reduce anemia risk. In contrast, this study utilized the DII to emphasize the importance of diet inflammation and holistic dietary regulation.

Although no prior studies have explicitly examined the relationship between the DII during pregnancy and third-trimester anemia, existing evidence suggests correlations between dietary inflammatory potential and hemoglobin levels. A prospective cohort study [49] on maternal anemia reported that higher DII scores were associated with lower third-trimester hemoglobin values (β = −0.057, *p* = 0.011). Similarly, analyses from the NEHANS database indicated that an elevated DII increases anemia susceptibility in the general population (OR = 1.06) [50]. Unlike previous studies, our research, based on a rigorously designed cohort excluding individuals with acute infections (which may transiently elevate inflammatory markers) and adjusting for baseline serum iron levels and iron supplementation, confirms the DII as an independent contributor to anemia risk during the third trimester.

Mechanistically, there exists a close interplay between diet, inflammation, and anemia. Elevated levels of pro-inflammatory biomarkers (e.g., IL-6, CRP) have been consistently linked to higher DII values in prior research [51,52]. Pro-inflammatory diets, as shown by high DII values, trigger systemic inflammation [53], which may mechanistically contribute to anemia. Research [54] highlights the critical link between anemia and inflammation. Under inflammatory conditions, macrophages release elevated levels of cytokines, stimulating the hepatic synthesis of hepcidin [55,56]. Hepcidin interacts with ferritin on enterocytes, triggering their internalization and proteolytic degradation and ultimately inhibiting iron absorption by intestinal epithelial cells [57,58]. This iron-restricted state exacerbates iron deficiency, increasing susceptibility to anemia in the third trimester. Concurrently, inflammation may shorten the erythrocytes’ lifespan through oxidative stress and eryptosis [19], further aggravating anemia. Moreover, chronic inflammation elevates the risks of gestational complications—including stillbirth, miscarriage, and placental abruption [59,60]—which may indirectly worsen anemia by compromising maternal nutritional status and physiological resilience. These pathways collectively explain, in part, how an elevated gestational DII increases the risk of third-trimester anemia, though more comprehensive investigations are required to fully characterize these mechanisms. Additionally, this study found a higher incidence of third-trimester anemia in passive smokers compared to the non-exposed group. However, the limited sample size of passive smokers in the anemia subgroup (*n* = 37) may have constrained the statistical power, necessitating cautious interpretation of this finding.

### 4.2. Research Significance of This Study

This study reveals a causal relationship between the DII in the first and second trimesters and third-trimester anemia, providing critical evidence for clinical practice and public health policies. At the clinical level, anti-inflammatory dietary interventions (e.g., increased intake of whole grains, dark leafy vegetables, and antioxidant-rich foods) [61] should be incorporated into comprehensive anemia management strategies, with individualized dietary guidance to reduce DII scores, particularly necessary for pregnant women who are not using iron supplements. At the public health level, integrating DII assessment tools [62] to dynamically monitor dietary inflammatory potential and promoting anti-inflammatory dietary concepts through community health education are recommended.

### 4.3. Limitations and Future Research

Although this study demonstrates a pro-inflammatory dietary model (assessed via the DII) elevates the risk of third-trimester anemia, several limitations should be acknowledged. First, in this study, we chose serum iron as an indicator of iron status due to its completeness and accessibility in the cohort database. However, the lack of anemia-related laboratory biomarkers such as serum ferritin (SF) may result in an inability to accurately reflect iron storage levels, which may affect the accuracy of our findings. Second, while pre-pregnancy BMI was included in analyses, gestational weight gain—a potential confounder—was not accounted for. Third, the self-administered nature of dietary questionnaires may introduce recall or reporting bias, which is a limitation of this study.

Future studies with expanded cohorts and comprehensive biomarker profiling are warranted to explore the relationships of the DII with anemia severity, along with conducting multi-ethnic, multi-regional, multi-center studies to validate the generalizability of the findings. The further refinement of dietary assessment methods and development of a rapid DII evaluation tool tailored for pregnancy are also needed.

## 5. Conclusions

There was a strong positive correlation between the gestational DII and anemia risk in the third trimester, indicating that pro-inflammatory diets during pregnancy are strongly associated with an elevated risk of this condition. Furthermore, this cause–effect relationship was more pronounced in participants who did not receive iron supplementation. Dietary interventions aimed at avoiding pro-inflammatory dietary patterns during pregnancy may reduce anemia risk in the third trimester, thereby improving maternal and newborn health. The conclusions of this study require cautious interpretation. Although the longitudinal design supports a temporal relationship between the DII and anemia risk, potential biases may arise from recall bias in dietary assessments, residual confounding (e.g., missing biomarkers), and sample size limitations. Future studies integrating biomarker-validated diets and multicenter cohorts are needed to confirm causality between dietary inflammation and anemia during pregnancy.

## Figures and Tables

**Table 1:** Comparison of baseline characterization by three groups of first–second-trimester average DII.

Variable	Total (*n* = 545)	Anti-Inflammatory Diet Group (*n* = 136)	Intermediate Group (*n* = 273)	Pro-Inflammatory Diet Group (*n* = 136)	*p* ^1^
Baseline serum iron	8.76 ± 0.85	8.69 ± 0.86	8.76 ± 0.84	8.81 ± 0.87	0.502
Maternal age, *n* (%)					0.869
<30 years	310 (56.88)	77 (56.62)	158 (57.88)	75 (55.15)	
≥30 years	235 (43.12)	59 (43.38)	115 (42.12)	61 (44.85)	
Occupation, *n* (%)					0.570
Employed	479 (87.89)	123 (90.44)	238 (87.18)	118 (86.76)	
Unemployed	66 (12.11)	13 (9.56)	35 (12.82)	18 (13.24)	
Pre-pregnancy BMI, *n* (%)					0.803
<24 kg/m^2^	478 (87.71)	121 (88.97)	237 (86.81)	120 (88.24)	
≥24 kg/m^2^	67 (12.29)	15 (11.03)	36 (13.19)	16 (11.76)	
Education, *n* (%)					0.692
Below high school	69 (12.66)	20 (14.71)	32 (11.72)	17 (12.50)	
High school and above	476 (87.34)	116 (85.29)	241 (88.28)	119 (87.50)	
Parity, *n* (%)					0.322
0	330 (60.55)	89 (65.44)	164 (60.07)	77 (56.62)	
≥1	215 (39.45)	47 (34.56)	109 (39.93)	59 (43.38)	
History of adverse pregnancy, *n* (%)					0.583
No	324 (59.45)	86 (63.24)	159 (58.24)	79 (58.09)	
Yes	221 (40.55)	50 (36.76)	114 (41.76)	57 (41.91)	
Passive smoking exposure, *n* (%)					0.088
No	442 (81.10)	119 (87.50)	216 (79.12)	107 (78.68)	
Yes	103 (18.90)	17 (12.50)	57 (20.88)	29 (21.32)	
Iron supplementation use, *n* (%)					0.562
Yes	231 (42.39)	63 (46.32)	112 (41.03)	56 (41.18)	
No	314 (57.61)	73 (53.68)	161 (58.97)	80 (58.82)	

Note: ^1^ Categorical and continuous variables were evaluated via the χ^2^ test and the Kruskal–Wallis test, respectively, to derive *p* values.

**Table 2:** Univariate analysis of third-trimester anemia risk factors.

Variable	Number of Participants	Number of Cases	Cumulative Incidence (%)	Statistic	*p*
Maternal age				χ^2^ = 0.13	0.721
<30 years	310	36	11.61		
≥30 years	235	25	10.64		
Pre-pregnancy BMI				χ^2^ = 3.47	0.063
<24	478	58	12.13		
≥24	67	3	4.48		
Occupation				χ^2^ = 0.33	0.563
Employed	66	6	11.48		
Unemployed	479	55	9.09		
Education				χ^2^ = 3.47	0.063
Below high school	67	3	15.94		
High school and above	478	58	10.50		
History of adverse pregnancy				χ^2^ = 0.39	0.531
No	324	34	10.49		
Yes	221	27	12.22		
Parity				χ^2^ = 1.88	0.211
0	330	32	9.70		
≥1	215	29	13.49		
Passive smoking exposure				χ^2^ = 5.13	0.023
No	442	56	12.67		
Yes	103	5	4.85		
Iron supplementation use				χ^2^ = 0.75	0.411
Yes	314	32	12.55		
No	231	29	10.19		
First–second-trimester average DII				χ^2^ = 7.67	0.022
Anti-inflammatory diet group	135	7	5.19 ^a^		
Intermediate group	275	33	12.00 ^a,b^		
Pro-inflammatory diet group	135	21	15.56 ^b^		

Note: Superscript letters (a, b) indicate the results of pairwise comparisons among groups using the Bonferroni correction. Groups sharing the same superscript letter (a, b) exhibit no statistically significant differences; those with different letters indicate significance.

**Table 3:** Relationship between first–second-trimester DII and anemia in third trimester (OR and 95% CI).

Duration of Pregnancy	Model I ^1^ OR (95% CI)	*p*	Model II ^2^ OR (95% CI)	*p*	Model III ^3^ OR (95% CI)	*p*
First-trimester DII	1.15 (0.99–1.33)	0.065	1.14 (0.98–1.32)	0.092	1.15 (0.99–1.34)	0.067
Second-trimester DII	1.23 (1.05–1.44)	0.009	1.23 (1.05–1.45)	0.009	1.26 (1.08–1.48)	0.004
First–second-trimester average DII (groups)						
Anti-inflammatory diet group (Ref)	1		1		1	
Intermediate group	2.53 (1.09–5.89)	0.031	2.53 (1.09–5.89)	0.030	2.82 (1.20–6.63)	0.017
Pro-inflammatory diet group	3.37 (1.38–8.21)	0.008	3.37 (1.38–8.21)	0.009	3.73 (1.50–9.25)	0.005

Note: ^1^ Model I represents the basic model; ^2^ Model II accounted for potential confounders, including age, pre-pregnancy BMI, education, occupation, parity, serum iron, and a history of adverse pregnancy outcomes; ^3^ Model III further adjusted for passive smoking exposure and iron supplementation based on Model II.

**Table 4:** Stratified analysis of iron supplementation, DII, and anemia risk.

DII Score	Non-Users of Iron Supplement		Users of Iron Supplements	
	aOR ^1^ (95% CI)	*p*	aOR ^1^ (95% CI)	*p*
Second-trimester DII	1.40 (1.12–1.78)	0.004	1.12 (0.90–1.41)	0.321
First–second-trimester average DII	1.62 (1.19–2.22)	0.002	1.19 (0.91–1.54)	0.199

Note: ^1^ Logistic regression models accounted for potential confounders: age, pre-pregnancy BMI, education, occupation, adverse pregnancy outcomes, parity, serum iron, passive smoking exposure, and iron supplementation.

## Data Availability

The data presented in this study are available on request from the corresponding author due to participant privacy concerns and legal restrictions related to the confidentiality agreements of the study cohort.
